# Transfemoral transcatheter aortic valve replacement for pure native aortic regurgitation: one-year outcomes of a single-center study

**DOI:** 10.1186/s12872-023-03329-1

**Published:** 2023-06-29

**Authors:** Hua-Jie Zheng, Yong-Bo Cheng, Chao-Jun Yan, De-Qing Lin, San-Jiu Yu, Jun Li, Ping He, Wei Cheng

**Affiliations:** Department of Cardiac Surgery, Southwest Hospital, Third Military Medical University (Army Medical University), No. 30, Gaotanyan, Shapingba District, Chongqing, 400038 China

**Keywords:** Transcatheter aortic valve replacement, Pure native aortic regurgitation, Venus A-Valve, Clinical outcomes

## Abstract

**Background:**

Evidence about safety and efficacy of transcatheter aortic valve replacement (TAVR) with the Venus A-Valve system (Venus Medtech, Hangzhou, China) remains limited for patients with pure native aortic regurgitation (PNAR).

**Objectives:**

The single-center study sought to report the one-year clinical outcomes of the Venus A-Valve in the treatment of PNAR.

**Methods:**

This study was a retrospective analysis of prospectively collected data. Data was from all consecutive patients who had PNAR and underwent TAVR with the Venus A-Valve system at our center from July 2020 and June 2021. Procedural and clinical outcomes up to one year were analyzed using Valve Academic Research Consortium-2 criteria.

**Results:**

A total of 45 consecutive patients with PNAR underwent transfemoral TAVR with the Venus A-Valve system. The Mean age was 73.5 ± 5.5 years and 26.7% were female. All the TAVR procedures were performed via transfemoral access. Implantations were successful in 44 cases (97.8%). Only one patient was converted to surgical aortic valve replacement. No patient died intraoperatively. No second valve was implanted. In-hospital mortality rate was 2.3%. The one-year all-cause mortality rate was 4.7% without cardiovascular related death. No patient had moderate or severe paravalvular leakage during follow-up. At one year, the mean pressure gradient was 8.8 ± 0.9 mmHg, and left ventricular ejection fraction increased to 61.5 ± 3.6%.

**Conclusions:**

This single-center study demonstrated the safety and efficacy of transfemoral TAVR with the Venus A-Valve in the treatment of patients with PNAR.

## Introduction

Aortic regurgitation (AR) affects about 13% of patients suffering from native valvular heart diseases [1, 2]. Pure native aortic regurgitation (PNAR) is usually characterized by leaflet degeneration, aortic root dilatation with aortic annulus enlargement, or both [3]. It is well known that surgical aortic valve replacement (SAVR) remains the standard treatment for patients with PNAR [4]. However, some patients have high surgical risks and postoperative mortality, resulting in many patients losing the chances of surgery. When left untreated, these patients face an annual mortality risk of 20% [5].

Transcatheter aortic valve replacement (TAVR) has been established as a treatment alternative for patients with symptomatic severe aortic stenosis (AS) who were at prohibitive or high-risk for SAVR [6]. Off-label uses of TAVR for treatment of PNAR has been reported with several devices. Overall outcomes of these studies were basically promising [7–9]. However, outcomes varied significantly between studies using different devices. The presence of large annular anatomy and the absence of valvular calcification have made the transcatheter treatment of PNAR challenging, mainly due to the risk of inadequate anchoring, prosthesis dislodgment, and residual paravalvular leak (PVL) after implantation [10, 11]. In the latest updated American College of Cardiology/American Heart Association guideline, it is stated that TAVR may be considered in experienced centers for selected patients with PNAR who are ineligible for SAVR [12].

The Venus A-Valve system (Venus Medtech, Hangzhou, China) is made of a self-expanding nitinol frame and tri-leaflet bovine pericardial valve [13]. TAVR with Venus A-Valve system to treat PNAR had encouraging results but the experience was still limited [8–14]. We now report the one-year outcomes of the single-center study with transfemoral implantation of the Venus A-Valve in patients with PNAR.

## Methods

### Study design

The study was a single-center retrospective analysis of prospectively collected data. Data was from all consecutive patients who had PNAR and underwent TAVR with the Venus A-Valve system at our center from July 2020 and June 2021. All patients finished one-year follow-up.

Every patient experienced clinical examination, laboratory test, transthoracic echocardiography and contrast-enhanced multidetector computed tomography (MDCT) at admission. The degree of AR is graded by measurement of the narrowest width of the proximal regurgitant jet (vena contracta) by Color Doppler [15]. A jet width < 0.3 cm indicates mild AR, while a width > 0.6 cm indicates severe AR.

All patients were evaluated by our heart team before operation and considered to be at prohibitive or high risk for SAVR. The inclusion criteria were: (1) age ≥ 60 with New York Heart Association (NYHA) functional class II-IV; (2) symptomatic PNAR with resting LVEF ≤ 50% or LVESD > 50 mm; (3) logistic EuroScore ＞ 20%. The exclusion criteria were: (1) patients with failed bioprosthetic surgical heart valves; (2) dimension of aortic root ＞ 50 mm; (3) diameter of aortic annulus ＞ 29 mm or ＜18 mm; (4) AS with peak aortic valve pressure gradient ＞ 20 mmHg or peak aortic velocity > 2.5 m/s; (5) serious comorbidities such as acute aortic dissection, severe coagulation disorder, and multiple organ failure.

Comprehensive clinical and echocardiographic assessments were scheduled before discharge, at 30 days, and at 6 and 12 months. Clinical follow-up was performed by direct or telephone interview according to each center^’^s practice. Data on baseline characteristics, operative details, postoperative outcomes, and follow-up information were collected prospectively and entered electronically in a dedicated Microsoft Access database.

### TAVR procedures

**Fig. 1:**
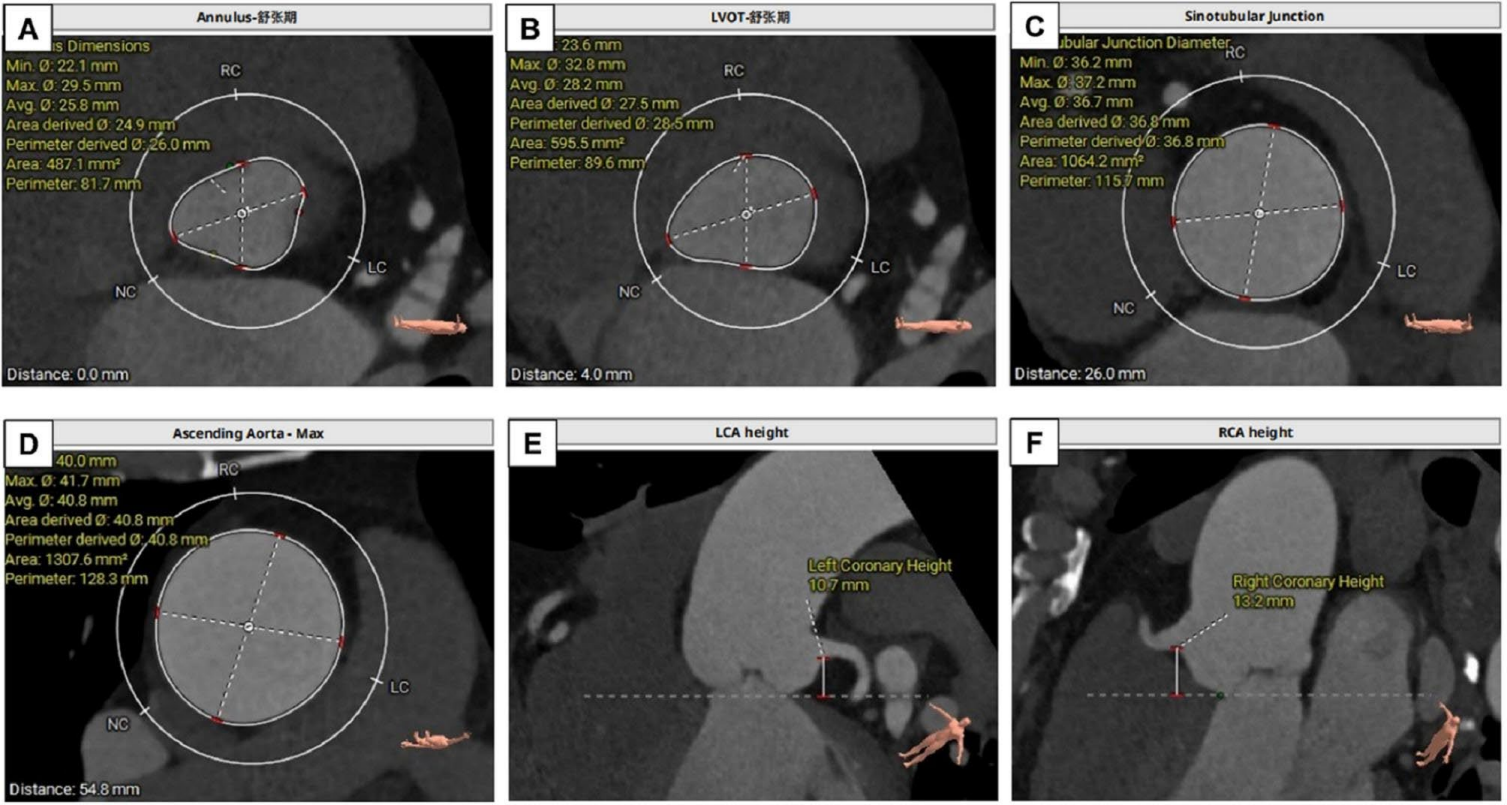
Aortic root measurements. **(A)** AA plane cross section; **(B)** LVOT plane cross section; **(C)** STJ plane cross section; **(D)** AAO plane cross section; **(E)** LCA height; **(F)** RCA height. AA, aortic annulus. LVOT, left ventricular outflow tract; STJ, sinotubular junction; AAO, ascending aorta; LCA, left coronary artery;RCA, right coronary artery

Pre-procedure aorta-iliac-femoral computed tomography was performed to evaluate the size of vessel caliber and feasibility of transfemoral approach. MDCT was used to assess the morphology of the aortic root (Fig. 1).

**Fig. 2:**
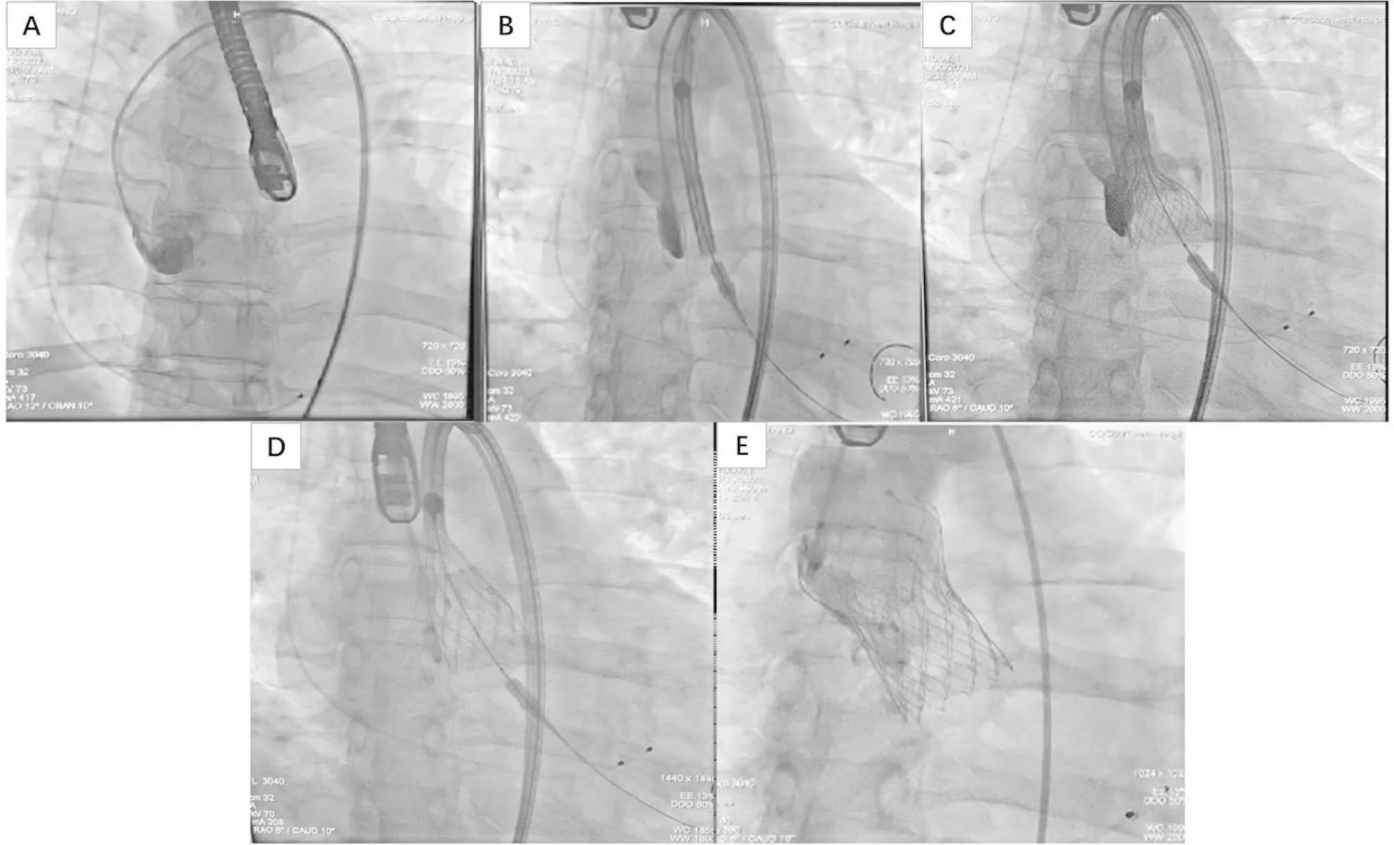
Fluoroscopic demonstration of transcatheter aortic valve replacement (TAVR) intraprocedural steps with the Venus A-Valve system. **(A)** the aortic Sinuses of Valsalva was positioned by angiography; **(B)** Transcatheter heart valve initial deployment position; **(C)** The valve was slowly deployed under rapid ventricular pacing; **(D)** implant height (3–5 mm depth); **(E)** A final deployment position

All procedures were performed under general anesthesia in the hybrid catheterization laboratory. The aortic Sinuses of Valsalva was positioned by angiography (Fig. 2A). The Venus A-Valve was carefully advanced, highly positioned (Fig. 2B), and slowly deployed under rapid ventricular pacing (180–200 beats/min) (Fig. 2C). The intended implantation depth is ranging from 3 to 5 mm below the virtual annular plane (Fig. 2D). After final deployment, rapid ventricular pacing was kept at 120–140 beats/min until delivery system removal. The final contrast injection showed proper prosthesis expansion, no central or paravalvular leak, and coronary arteries with adequate flow (Fig. 2E).

### Study endpoints

Procedural and clinical outcomes up to one year were analyzed using Valve Academic Research Consortium-2 (VARC-2) criteria [16]. The primary endpoint was the composite endpoint of device success, defined as: absence of procedural mortality, successful vascular access, delivery and deployment of the device, successful retrieval of the delivery system, correct final position of the device, proper functioning of the prosthetic heart valve (mean gradient < 20 mm Hg, peak velocity < 3 m/s, absence of moderate or severe AR), and no need for valve-in valve implantation or surgical conversion. Secondary endpoints were the other echocardiographic assessment of the valve and cardiac function.

### Statistical analysis

Normally distributed continuous variables were presented as mean ± standard deviation (SD), non-normally distributed variables as median and range, categorical variables as raw counts and percentages. Assessment of normality was performed using the Shapiro-Wilk test. Statistical analyses were performed using statistical analysis software (SPSS version 22.0, IBM, New York, NY).

## Results

### Baseline characteristics

Between July 2020 and June 2021, 133 consecutive patients who underwent transfemoral TAVR with the Venus A-Valve system at our center, of which 23 patients who had combined AS and AR. Among the remaining 110 patients, 65 had only AS; rest 45 had PNAR which was our study population. The mean age was 73.5 ± 5.5 years and 26.7% were female. One patient had congenital bicuspid aortic valve, while the others had tricuspid aortic valves. The mean risk score according to the logistic EuroSCORE (European System for Cardiac Operative Risk Evaluation) was 28.5 ± 7.5%, and 95.6% of the patients were in NYHA functional class ≥ III. The detailed description of patient characteristics was listed in Table 1.

**Table 1:** Baseline Characteristics

Characteristic	Value
Age (years)	73.5 ± 5.5
Female	12 (26.7)
Body mass index (kg/m^2^)	22.5 ± 3.5
Hypertension	22 (48.9)
Diabetes mellitus	12 (26.7)
Chronic pulmonary disease	19 (42.2)
Chronic renal dysfunction	10 (22.2)
Peripheral vascular disease	5 (11.1)
Prior cerebrovascular accident	8 (17.8)
Anemia	9 (20.0)
Atrial fibrillation	4 (8.9)
Coronary artery disease	7 (15.6)
Prior myocardial infarction	0
Prior PCI	5 (11.1)
Prior CABG	0
Prior mitral valve surgery	2 (4.4)
Prior permanent pacemaker implantation	2 (4.4)
Etiology	
Degenerative	18 (40.0)
Rheumatic	26 (57.8)
Bicuspid aortic valve	1 (2.2)
Aortic regurgitation	
≤ Moderate	0
Moderate to Severe	10 (22.2)
Severe	35 (77.8)
Mitral regurgitation ≥ moderate	16 (35.6)
Pulmonary hypertension	11 (24.4)
Logistic EuroSCORE, %	28.5 ± 7.3
NYHA functional class	
I	0
II	2 (4.4)
III	14 (31.1)
IV	29 (64.4)

Values are expressed as mean ± standard deviation or number (%). NYHA = New York Heart Association; EuroSCORE = European System for Cardiac Operative Risk Evaluation; PCI = percutaneous coronary intervention; CABG = coronary artery bypass graft surgery.

### Procedural details and in-hospital outcomes

All the TAVR procedures were performed via transfemoral access. The procedure was successful in 97.8% (44/45). One patient was converted to SAVR because of valve embolism into aortic arch, and recovered well after the aortic valve and hemi-arch replacement. In our study, the 26-mm, 29-mm, and 32-mm valve was implanted in 3 patients (6.8%), 23 patients (52.2%), and 18 patients (40.9%), respectively. The average procedural time were 71.1 ± 16.6 min. No patient died intraoperatively. One patient with a bicuspid aortic valve had moderate PVL, and died of low cardiac output syndrome after refusing further treatment on postoperative day 5. The other patients developed PVL no more than mild degree. Additionally, no balloon post-dilation was performed in any patient. No coronary obstruction, prosthesis malposition, annular rupture or new cerebrovascular accident occurred. No second valve or new permanent pacemaker was implanted. Two patients needed blood transfusion due to preprocedural anemia. According to the Acute Kidney Injury (AKI) Network classification [14], five patients with prior chronic renal dysfunction developed stage 1 AKI, while the renal function gradually recovered before discharge. In-hospital mortality rate was 2.3%. The mean duration of intensive care unit (ICU) stay and postoperative hospital stay was 2.5 ± 0.5 and 4.0 ± 1.5 days, respectively. Procedural details and in-hospital outcomes were listed in Table 2.

**Table 2:** Procedural Details and In-Hospital Outcomes

Parameter	Value
Aortic annulus diameter	n = 45
MDCT perimeter-derived, mm	26.3 ± 2.5
MDCT area-derived, mm	26.0 ± 2.4
STJ diameter, mm	38.2 ± 5.5
LCA ostium height, mm	13.5 ± 3.3
RCA ostium height, mm	15.2 ± 5.3
Ascending aortic diameter, mm	42.5 ± 5.9
Aortic root diameter, mm	39.8 ± 5.5
Successful implantation	44 (97.8)
Conversion to SAVR	1 (2.2)
THV size	n = 44
26-mm	3 (6.8)
29-mm	23 (52.2)
32-mm	18 (40.9)
Procedure time, min	71.1 ± 16.6
Contrast agent, mL	85.5 ± 22.5
Balloon post-dilation	0
Combined PCI	0
Coronary obstruction	0
Prosthesis malposition	0
Annulus rupture	0
New cerebrovascular events	0
Second valve implantation	0
New permanent pacemaker implantation	0
Transfusion	2 (4.7)
Acute kidney injury	0
Stage 1	5 (11.6)
Stage 2 or 3	0
Central aortic prosthetic regurgitation	n = 44
None or trace	43 (100)
≥ Mild	0
Procedure-related death	0
In-hospital mortality	1 (2.3)
ICU stay, days	2.5 ± 0.5
Post operation In-hospital stay, days	4.0 ± 1.5

Values are expressed as mean ± standard deviation or number (%). MDCT, multidetector computed tomography; STJ, sinotubular junction; LCA, left coronary artery; RCA, right coronary artery; SAVR, surgical aortic valve replacement; THV, transcatheter heart valve; PCI = Conversion to conventional surgery aortic valve replacement; ICU = intensive care unit.

### One-year outcomes

All patients were followed up postoperatively up to one year by telephone or direct interview (100% completed / no lost at follow-up). The one-year all-cause mortality rate was 4.7% without cardiovascular related death. One patient died of severe acute pancreatitis 9 months after discharge, and the other patient died of a car accident 11 months after discharge. All of the remaining 41 patients fortunately survived. Only one patient was in NYHA functional class III, who developed a third-degree atrioventricular block 10 months after discharge and received permanent pacemaker implantation. No patient experienced new myocardial infarction, new cerebrovascular accident, valve thrombosis, or valve-related reintervention. Detailed outcomes at one-year follow-up were listed in Table 3.

**Table 3:** One-Year Clinical Outcomes

Parameter	Value
All-cause mortality	2 (4.7)
Cardiovascular mortality	0
NYHA functional class	n = 41
I or II	40 (97.6)
III	1 (2.4)
IV	0
New permanent pacemaker implantation	1 (2.4)
New Myocardial infarction	0
New cerebrovascular accident	0
Valve thrombosis	0
Valve-related reintervention	0
Central aortic prosthetic regurgitation	n = 41
None or trace	41 (100)
≥ Mild	0
Mitral regurgitation ≥ moderate	3 (7.3)

Values are expressed as mean ± standard deviation or number (%). NYHA = New York Heart Association.

### Echocardiography assessments

**Fig. 3:**
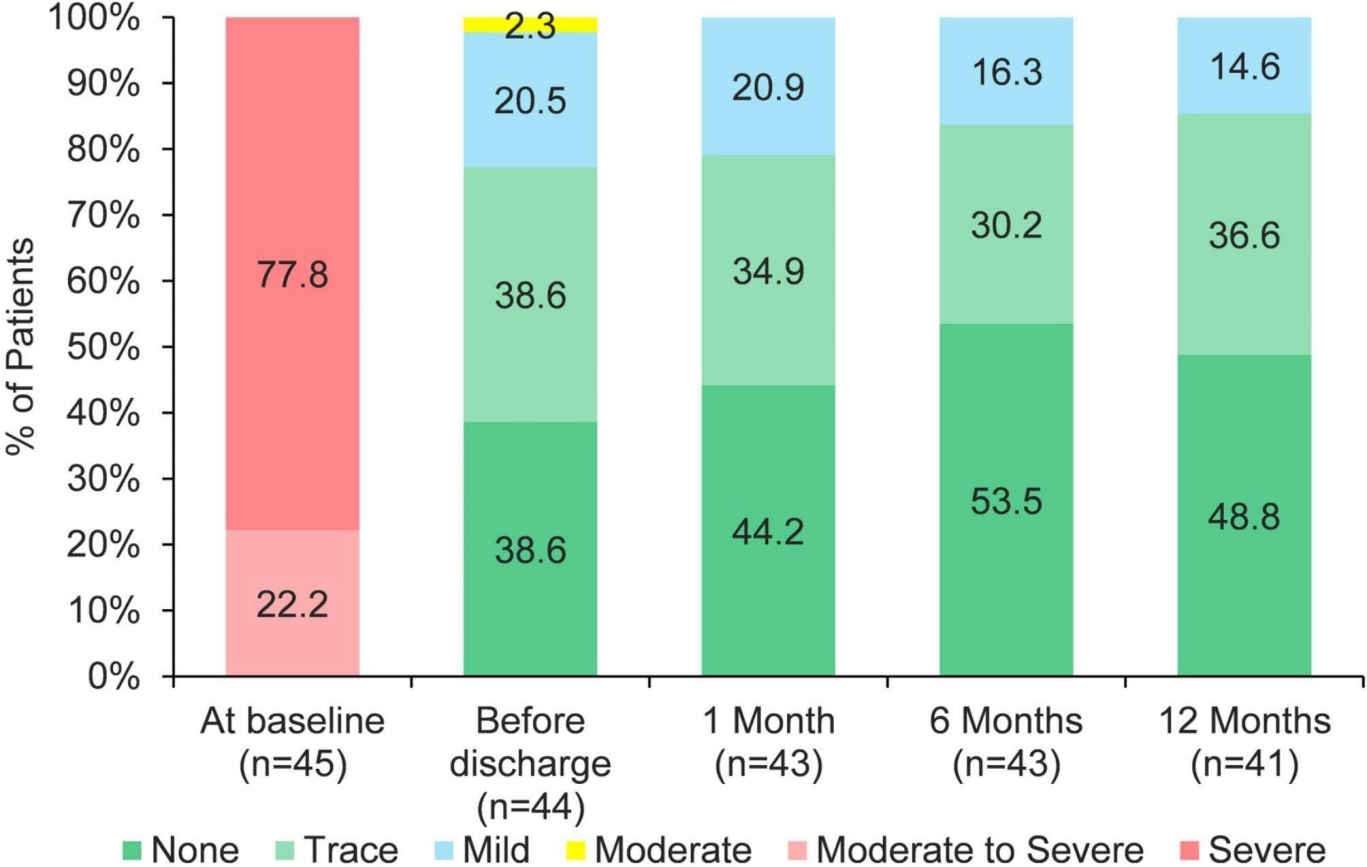
The incidence of aortic regurgitation at baseline and paravalvular leak (PVL) during one-year follow-up

**Fig. 4:**
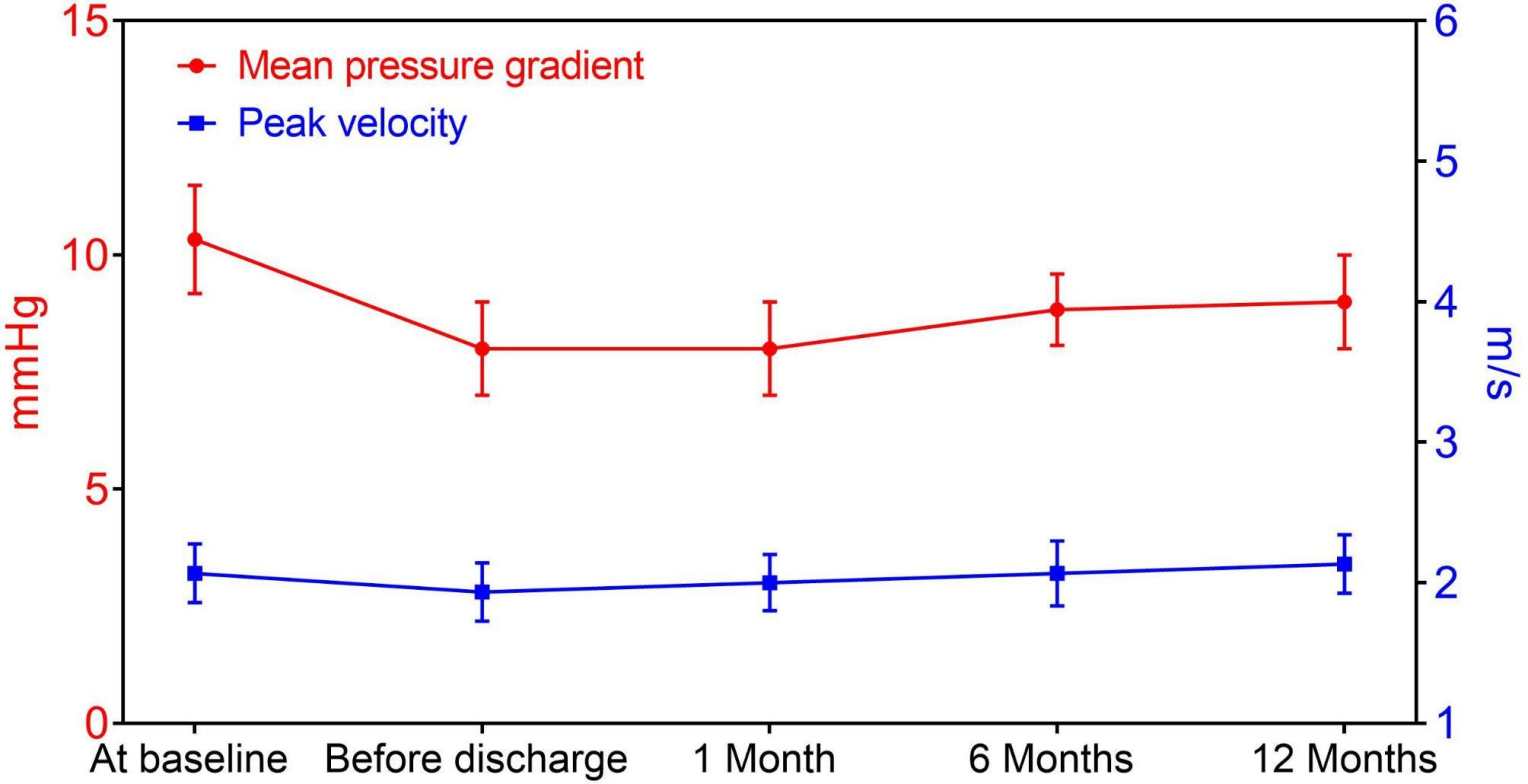
Changes of mean pressure gradient and peak velocity from baseline to one-year follow-up

**Fig. 5:**
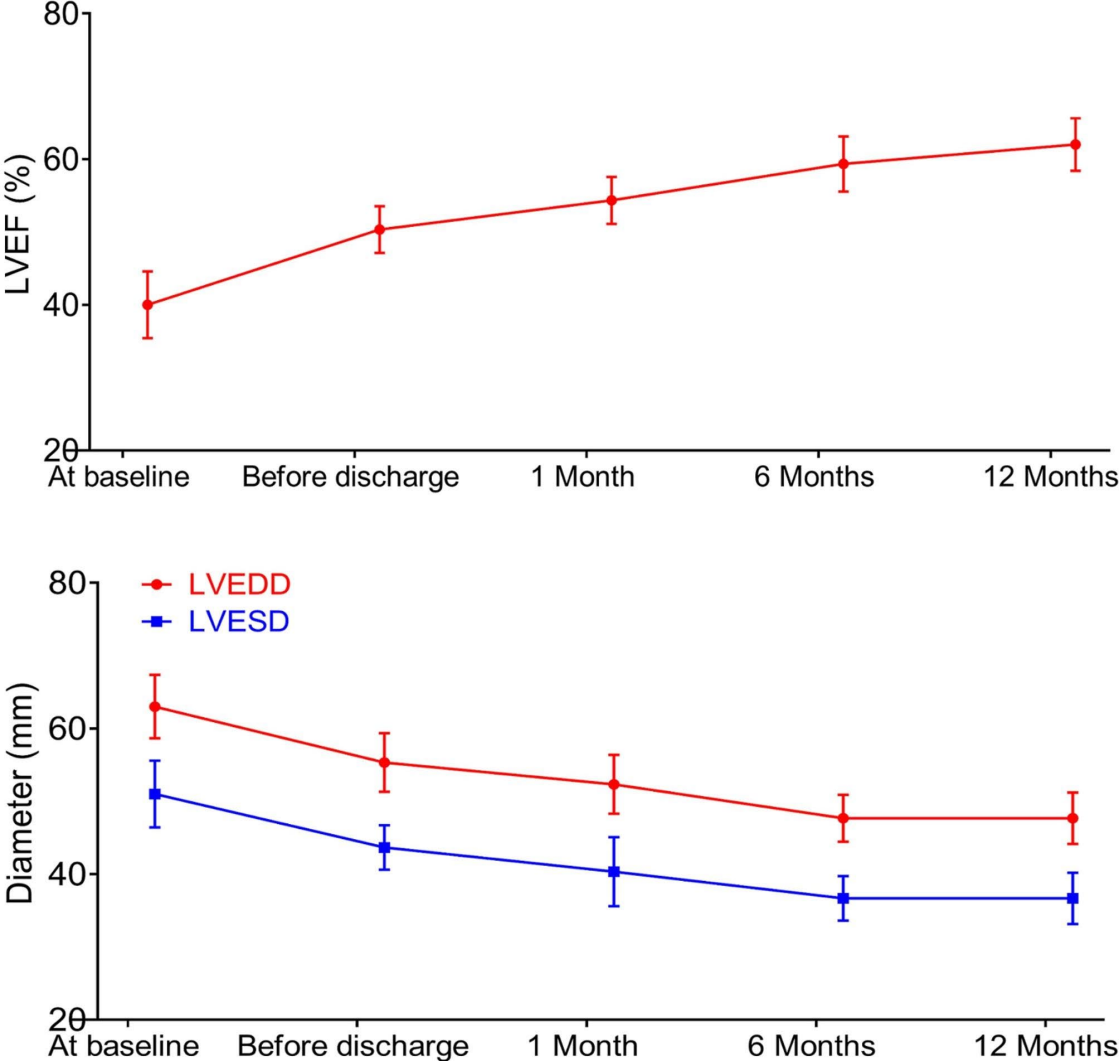
Changes of LVEF, LVEDD and LVESD from baseline to one-year follow-up. LVEF, left ventricular ejection fraction; LVEDD, left ventricular end-diastolic dimension; LVESD, left ventricular end-systolic dimension

The degree of AR at baseline and the PVL during follow-up were listed in Fig. 3. No patient had moderate or severe PVL at one month, six months and twelve months. The mean pressure gradient was 8.1 ± 1.2 mmHg at one month, 8.8 ± 0.9 mmHg at one year (Fig. 4). The aortic valve peak velocity remained stable at one month, six months and twelve months. The LVEF significantly increased from 41.5 ± 4.6% at baseline to 61.5 ± 3.6% at one year (Fig. 5). Additionally, significant decreases were observed in LVEDD (from 62.1 ± 4.9 mm at baseline to 46.0 ± 3.6 mm at one year) and LVESD (from 51.2 ± 4.8 mm at baseline to 36.9 ± 3.7 mm at one year).

## Discussion

This single-center study confirmed the feasibility of transfemoral TAVR with the Venus A-Valve in patients with PNAR. The procedure was successful in 97.8%. No patient died intraoperatively. No second valve was implanted in any case. Echocardiographic measurements showed adequate hemodynamic function without PVL > moderate degree, significant improvement in LVEF and decreased mean pressure gradient during one-year follow-up.

The Venus A-Valve is a self-expanding frame, porcine pericardial valve with supra-annular leaflets [17]. The prosthesis is available in 23-mm, 26-mm, 29-mm and 32-mm sizes and has three radiopaque markers 6 mm from the inflow to aid in precise positioning. The implantation depth is ranging from 3 to 5 mm below the virtual annular plane. At 3 to 5 mm, the outward radial forces of the aortic valve stent frame and the annular coverage of the conforming frame are optimal and should provide excellent results.

The Venus A-Valve system has some unique advantages over other self-expanding valves [18]. First, it can be fully retrieved if there is significant residual PVL or the prosthesis position is not proper; second, it can correct the deployment position in real time; third, it can check the stability of the prosthesis during operation. Despite these advantages, in our early procedures, one patient was converted to SAVR due to valve embolism into the aortic arch. Of note, the size, positioning, and anchoring of the prosthetic valve may be important factors in this valve embolization event. The increased stroke volume caused by significant AR and the low implantation height due to the absence of fluoroscopic calcific landmarks may also be the important factors. To prevent this complication, we updated our protocol in subsequent cases, such as a prolonged observation time without removing the wires to avoid valve inversion in case of embolization and to allow subsequent balloon recapture maneuvers. Since then, valve embolization never happened again.

The diameter of aortic annulus for sizing the prosthesis was calculated by the perimeter and area of the native aortic annulus [19]. It was necessary to have a prosthesis/annulus oversizing of 15–25% to minimize the risk of insufficient prosthesis anchoring and PVL [20]. Oversizing beyond 25% was not recommended due to the risk of annular rupture and conduction system abnormality. In our experience, 10–20% oversizing of the native aortic annulus was recommended for the Venus A system. Anchoring of transcatheter heart valve in PNAR relied on aortic annulus, left ventricular outflow tract (LVOT), sinotubular junction (STJ), and thickening leaflet [21]. Aortic annulus and LVOT in general crucial for stable anchoring of a transcatheter heart valve. STJ may provide an anchoring for the “crown” of the prosthetic valve and avoid it slipping down. Moreover, the thickening leaflets provides the much greater friction between the native valve and prosthetic valve frame.

The choice of prosthesis can help avoid the most common complications of TAVR in the treatment of patients with PNAR, such as significant PVL and valve migration or embolization. In absence of significant aortic valve calcification for anchoring, newer generation prostheses performed better than old generation valves [22]. In addition, the repositionability and aortic stabilization of self-expanding valves (SEV) may be an attractive option, while balloon-expandable valves (BEV)balloon-expandable valves (BEV) designs with prominent outer skirts and the ability to oversize significantly may also make them reasonable alternatives. Of course, dedicated prostheses for PNAR have been developed with native leaflet anchoring design, including the J-Valve and Jena Valve, and the success rate of the procedure has increased to over 90% [23, 24]. In our study transfemoral TAVR with the Venus-A system could achieve a similar procedural success rate.

Based on our experience, there are several technical points that should be noted. First, accurately determining the size of the prosthetic valve is critical. Second, two pigtail catheters should be positioned in the aortic sinuses and transesophageal echocardiography is essential to guide the valve implantation. Third, rapid ventricular pacing is necessary to reduce stroke volume, stabilize the annulus, and limit prosthesis motion. Last but not least, cardiopulmonary bypass should be prepared in some special cases.

### Study limitations

This study included a relatively small number of patients. The longest follow-up period was limited to one year. Further research with a larger patient population and longer follow-up duration are scheduled.

## Conclusion

This single-center study demonstrated the feasibility of transfemoral TAVR with the Venus A-Valve in patients with PNAR. Procedural and one-year follow-up results were promising. Continued observation is now warranted to confirm persistent valve function during long-term follow-up. We also need to further develop devices specifically for the treatment of PNAR and gain surgical experience to provide better clinical outcomes in the future.

## Data Availability

The data that support the findings of this study are available from the corresponding authors upon reasonable request.
